# The detectable anti-interferon-γ autoantibodies in COVID-19 patients may be associated with disease severity

**DOI:** 10.1186/s12985-023-01989-1

**Published:** 2023-02-21

**Authors:** Po-Ku Chen, Kai-Jieh Yeo, Shih-Hsin Chang, Tsai-Ling Liao, Chia-Hui Chou, Joung-Liang Lan, Ching-Kun Chang, Der-Yuan Chen

**Affiliations:** 1 Rheumatology and Immunology Center, China Medical University Hospital, No. 2, Yude Road, Taichung, 40447 Taiwan; 2 College of Medicine, China Medical University, Taichung, Taiwan; 3 Translational Medicine Laboratory, Rheumatology and Immunology Center, Taichung, Taiwan; 4 Ph.D. Program in Translational Medicine and Rong Hsing Research Center for Translational Medicine, National Chung Hsing University, Taichung, Taiwan; 5 Department of Medical Research, Taichung Veterans General Hospital, Taichung, Taiwan; 6 Division of Infection, China Medical University Hospital, Taichung, Taiwan; 7 Rheumatic Diseases Research Center, China Medical University Hospital, Taichung, Taiwan

**Keywords:** SARS-CoV-2, Anti-interferon-γ auto-Abs, Titers, Disease severity, COVID-19

## Abstract

**Background:**

Neutralizing anti-interferon (IFN)-γ autoantibodies are linked to adult-onset immunodeficiency and opportunistic infections.

**Methods:**

To explore whether anti-IFN-γ autoantibodies are associated with disease severity of coronavirus disease 2019 (COVID-19), we examined the titers and functional neutralization of anti-IFN-γ autoantibodies in COVID-19 patients. In 127 COVID-19 patients and 22 healthy controls, serum titers of anti-IFN-γ autoantibodies were quantified using enzyme-linked immunosorbent assay, and the presence of autoantibodies was verified with immunoblotting assay. The neutralizing capacity against IFN-γ was evaluated with flow cytometry analysis and immunoblotting, and serum cytokines levels were determined using the MULTIPLEX platform.

**Results:**

A higher proportion of severe/critical COVID-19 patients had positivity for anti-IFN-γ autoantibodies (18.0%) compared with non-severe patients (3.4%, *p* < 0.01) or healthy control (HC) (0.0%, *p* < 0.05). Severe/critical COVID-19 patients also had higher median titers of anti-IFN-γ autoantibodies (5.01) compared with non-severe patients (1.33) or HC (0.44). The immunoblotting assay could verify the detectable anti-IFN-γ autoantibodies and revealed more effective inhibition of signal transducer and activator of transcription (STAT1) phosphorylation on THP-1 cells treated with serum samples from anti-IFN-γ autoantibodies-positive patients compared with those from HC (2.21 ± 0.33 versus 4.47 ± 1.64, *p* < 0.05). In flow-cytometry analysis, sera from autoantibodies-positive patients could also significantly more effectively suppress the STAT1 phosphorylation (median,67.28%, interquartile range [IQR] 55.2–78.0%) compared with serum from HC (median,106.7%, IQR 100.0–117.8%, *p* < 0.05) or autoantibodies-negative patients (median,105.9%, IQR 85.5–116.3%, *p* < 0.05). Multivariate analysis revealed that the positivity and titers of anti-IFN-γ autoantibodies were significant predictors of severe/critical COVID-19. Compared with non-severe COVID-19 patients, we reveal that a significantly higher proportion of severe/critical COVID-19 patients are positive for anti-IFN-γ autoantibodies with neutralizing capacity.

**Conclusion:**

Our results would add COVID-19 to the list of diseases with the presence of neutralizing anti-IFN-γ autoAbs. Anti-IFN-γ autoantibodies positivity is a potential predictor of severe/critical COVID-19.

**Supplementary Information:**

The online version contains supplementary material available at 10.1186/s12985-023-01989-1.

## Background

Interferon (IFN)-γ, a type II IFN (IFN-II) and pro-inflammatory cytokine of innate immunity, is essential for the host’s defense against infection with intracellular pathogens [1, 2]. Although the biological mechanism of autoantibody formation against IFN-γ (anti-IFN-γ autoAbs) remains unclear, several studies have shown that these autoAbs have an inhibitory effect on IFN-γ signal transduction [3, 4]. Thus, the neutralizing anti-IFN-γ autoAbs are recognized as a cause of adult-onset immunodeficiency (AOID) and associated with increased risks of infection such as disseminated nontuberculous mycobacteria (NTM), non-typhoid *Salmonella*, *Cryptococcus*, and varicella-zoster virus (VZV), particularly in Asian populations [3–9]. Hong et al. revealed that anti-IFN-γ autoAbs levels were strongly associated with the severity of infections and likely related to their biological activity [10]. During this coronavirus disease 2019 (COVID-19) pandemic, the relationship between anti-IFN-γ autoAbs and the severity of COVID-19 is worth investigation.

Globally, more than 630 million people had been infected with severe acute respiratory syndrome coronavirus 2 (SARS-CoV-2), and more than 6.5 million people had died of COVID-19 as of October 2022. About 10-20% of COVID-19 patients developed severe or life-threatening complications [11, 12]. The Infection with SARS-CoV-2, like other viral infections, can trigger vigorous innate and adaptive immune responses to enhance viral clearance, and an aberrant adaptive immunity to SARS-COV-2 infection may result in autoimmune antibody formation [13–15]. Recently, Bastard et al. demonstrated that 10.2% of 987 life-threatening COVID-19 pneumonia patients had neutralizing autoAbs against type-I IFNs (IFNs-I), in contrast to the absence of autoAbs in asymptomatic or mild COVID-19 patients [16]. Chang et al. also revealed the development of IgG autoAbs, including those against IFNs-I, in a significant proportion of severe COVID-19 patients [17]. Given that the clinical outcome of COVID-19 is heterogeneous, and 10-20% of patients develop severe/critical illness [11, 18], it is helpful to stratify patients early in their disease course based on the emergence of neutralizing autoAbs against IFNs.

This pilot study aimed to assess the prevalence of anti-IFN-γ autoAbs in COVID-19 patients using ELISA and verify their presence using immunoblotting. We then investigated the blocking effect of anti-IFN-γ autoAbs on the IFN-γ signaling pathway, the STAT1 transactivation. We finally identified the potential markers predictive of COVID-19 severity.

## Methods

### Patients and study design

In this prospective and cross-sectional study, Taiwan Health Research Institute sponsored 102 random serum samples obtained from Chinese patients with laboratory-confirmed COVID-19. Twenty-five serum samples obtained from 25 donors with laboratory-confirmed COVID-19 were purchased from BocaBiolistics (Pompano Beach, FL, USA) (SOP 10-00414 Rev E [De-linking specimens]) as the other independent ethnic cohort. Patients’ data, including demographics, clinical manifestations, and comorbidities, were provided along with the specimens. All the COVID-19 patients’ specimens investigated in the present study were obtained between April 2020 and May 2021, during which time Alpha, Beta, and Gamma variants were the major Variants of Concern. None of them had received exogenous IFN-γ treatment. COVID-19 was confirmed if a nasal or pharyngeal swab specimen tested positive for COVID-19 using the polymerase-chain-reaction assay. The severity of COVID-19 patients was stratified according to the US National Institutes of Health (NIH) classification [19], and mild or moderate COVID-19 was considered non-severe. Besides, the timing of blood collection from the COVID-19 patients was not all during the acute infection phase. Among the COVID-19 patients, 81 (63.8%) had their blood samples taken at the time of hospital admission, and 46 (36.2%) at the time of discharge.

Twenty-two healthy Chinese volunteers without autoimmune diseases or previous SARS-CoV-2 infection were enrolled as healthy control (HC) subjects. The Institutional Review Board approved this study (CMUH110-REC1-086), and each HC participant’s written consent was obtained according to the Declaration of Helsinki.

### Determination of serum titers of anti-IFN-γ autoAbs with ELISA

Ten milliliters of whole blood were collected in BD Vacutainer® Serum Tubes (BD Biosciences, San Jose, CA, USA) and centrifuged at 2,000 rpm for 10 min. According to the manufacturer’s instructions, serum titers of anti-IFN-γ autoAbs were determined with ELISA (Cell Sciences, Newburyport, MA, USA). A “positive” ELISA result was defined as an anti-IFN-γ autoAbs titer ≥30 U/ml, and the cut-off value was defined as the mean value of HC subjects plus 3-fold standard deviations (SDs).

### Validation of the presence of anti-IFN-γ autoAbs with immunoblotting

To further confirm the presence of anti-IFN-γ autoAbs, immunoblotting was performed as described in previous reports [20, 21]. Briefly, recombinant human (rh)IFN-γ (10ng) (R&D Systems, Minneapolis, MN, USA) was separated using SDS-polyacrylamide gel electrophoresis and then transferred to a polyvinylidene difluoride membrane (Millipore, Billerica, MA, USA). After blocking with 5% bovine serum albumin (BSA) and then incubating with a serum sample from patients or HCs (1,000 dilutions) with or without anti-IFN-γ autoAbs, the immunoblots were hybridized with HRP-conjugated goat anti-human IgG (Jackson Immunology Research Inc, West Grove, PA, USA). The immunoreactive bands to IFN-γ expression were visualized using an enhanced chemiluminescence detection system (Millipore, Billerica, MA, USA).

### Functional evaluation of anti-IFN-γ autoAbs with flow cytometry analysis

The blocking ability of anti-IFN-γ autoAbs was assessed according to their effect on IFN-γ-induced signal transducer and activator of transcription1 (STAT1) phosphorylation in the human monocytic cell lines (THP-1, BCRC 60430, the Bioresource Collection and Research Center, Taiwan). Serum samples (10%) were incubated with or without 20 ng/ml rhIFN-γ at 37 °C for 30 min. The THP-1 cells were stimulated with IFN-γ (20ng/ml) and serum samples from COVID-19 patients or HCs [20–22]. The cells were washed twice with PBS and fixed with absolutely ice-cold methanol on ice for 10 min. After washing, THP-1 cells were stained with phycoerythrin (PE) anti-human phospho-STAT1 (pY701) monoclonal antibody (clone A17012A, BD Biosciences San Diego CA, USA) for 30 min at room temperature. The population of PE-pSTAT1 was analyzed by flow-cytometer with FlowJo version 7.6 software.

### Determination of serum levels of cytokines

Serum levels of IFN-α2, IFN-γ, Interleukin (IL)-6, and tumor necrosis factor (TNF)-α were determined with magnetic multiplex particle-based assay (Multiplex MAP kits, EMD Millipore, Billerica, MA, USA) according to the manufacturer’s instructions.

### Statistical analysis

The results were presented as the mean ± standard deviation (SD), the standard error of the mean (SEM), or the median (interquartile range, IQR). We performed a chi-squared test to examine the between-group difference of categorical variables. The Kruskal–Wallis test with a post-hoc Dunn’s test was used to compare different groups. The missing values were excluded from the statistical analysis. We also constructed both univariate and multiple logistic regression models to identify factors predictive of COVID-19 severity. A two-sided probability of less than 0.05 was considered significant. The statistical power is calculated using two-group (39 patients with severe COVID-19 and 88 patients with non-severe COVID-19) mean values of anti-IFN-γ autoantibodies, with a mean difference ranging from 9 to 11 and an average standard deviation of 20. The range of power is from 0.665 to 0.831.

## Results

### Clinical characteristics of COVID-19 patients

As illustrated in Table 1, patients with severe/critical COVID-19 were older than non-severe COVID-19 patients or HC participants (both *p* < 0.05). Fever was the most common manifestation in COVID-19 patients (75.6%) and a higher proportion of severe/critical COVID-19 patients had fever (87.2% vs. 70.5%, *p* <0.05) and pulmonary involvement (92.3% vs. 31.8%, *p* <0.001) compared with non-severe COVID-19 patients. As illustrated in Additional file 2: Table S1, a significantly higher proportion of males was observed in the Hispanic (64.0%) and Chinese patients (52.9%) compared with Chinese HC participants (27.3%, *p* <0.01 and *p* <0.05, respectively).

**Table 1:** Demographic data, clinical manifestations, and laboratory findings in severe/critical or non-severe patients with COVID-19 and healthy subjects

	Severe/critical COVID-19 (n = 39)	Non-severe COVID-19 (n = 88)	Healthy control (HC) (n = 22)
Age at study entry, years	52.3 ± 14.1*^,^^**#**^	45.2 ± 16.6	41.9 ± 12.3
Age ≧ 50 years	27 (69.2%)^$$$,**+++**^	37 (42.1%)^**+**^	5 (22.7%)
Male proportion, n (%)	20 (51.3%)	50 (56.8%)	6 (27.3%)
Fever, n (%)	34 (87.2%)^$^	62 (70.5%)	NA
Fatigue or myalgia, n (%)	17 (43.6%)	41 (46.6%)	NA
Skin rash, n (%)	2 (5.1%)	5 (5.7%)	NA
Sore throat, n (%)	8 (20.5%)	19 (21.6%)	NA
Liver dysfunction^a^	3 (7.7%)	7 (8.0%)	NA
Lung involvement^b^, n (%)	36 (92.3%)^$$$^	28 (31.8%)	NA
Gastrointestinal symptoms^c^, n(%)	10 (25.6%)	16 (18.2%)	NA
Headache, n (%)	7 (17.9%)	10 (11.4%)	NA
Dysosmia, n (%)	4 (10.3%)	7 (8.0%)	NA
Anti-IFN-γ autoAb (+), %	7 (18.0%)**^,^^**#**^	3 (3.4%)^,^	0 (0.0%)
Anti-IFN-γ autoAb level, U/mL	5.01 (0.4–18.9)^**#**^	1.33 (0.06–8.05)	0.44 (0.04–8.7)
IFN-γ levels, pg/mL	1.9 (0.7–3.7)^###^	2.0 (0.7–3.7)^###^	0.7 (0.5–0.8)
IFN-α2 levels, pg/mL	4.4 (2.3–19.8)	4.4 (2.9–13.7)	4.8 (2.8–11.1)
IL-6 levels, pg/mL	3.6 (0.9–26.7)^###^	2.1 (0.5–12.7)^###^	0.3 (0.2–0.7)
TNF-α levels, ng/mL	14.8 (10.3–21.9)	14.8 (9.1–23.0)	12.9 (9.7–16.3)
*Comorbidities*			
Hypertension, n (%)	13 (33.3%)	21 (23.9%)	0 (0.0%)
Diabetes mellitus, n (%)	6 (15.4%)	9 (10.2%)	0 (0.0%)

Data were expressed as mean ± SD, number (%), or median (25th–75th quartile range)

COVID-19: coronavirus disease 2019; IFN: interferon; IL: interleukin; TNF-α: tumor necrosis factor-α; NA: not applicable

^a^The presence of a twofold or more increase in alanine transaminase (ALT) that exceeded the upper limit of normal value (40 U/L)

^b^The presence of pneumonitis or interstitial lung change

^c^The presence of nausea, vomiting, or diarrhea

*p < 0.05, **p < 0.01, ***p < 0.001, versus non-severe COVID-19 patients, as determined by the Kruskal–Wallis test using a post-hoc Dunn’s test

^#^*p* < 0.05, ^###^*p* < 0.001, versus HC subjects, as determined by the Kruskal–Wallis test using a post-hoc Dunn’s test

^$^*p* < 0.05, ^$$$^*p* < 0.001, versus non-severe COVID-19 patients, as determined by Chi-square text

^+^*p* < 0.05, ^+++^*p* < 0.001, versus HC subjects, as determined by Chi-square text

### Increased prevalence and titers of anti-IFN-γ autoAbs in severe/critical COVID-19 patients

**Fig. 1:**
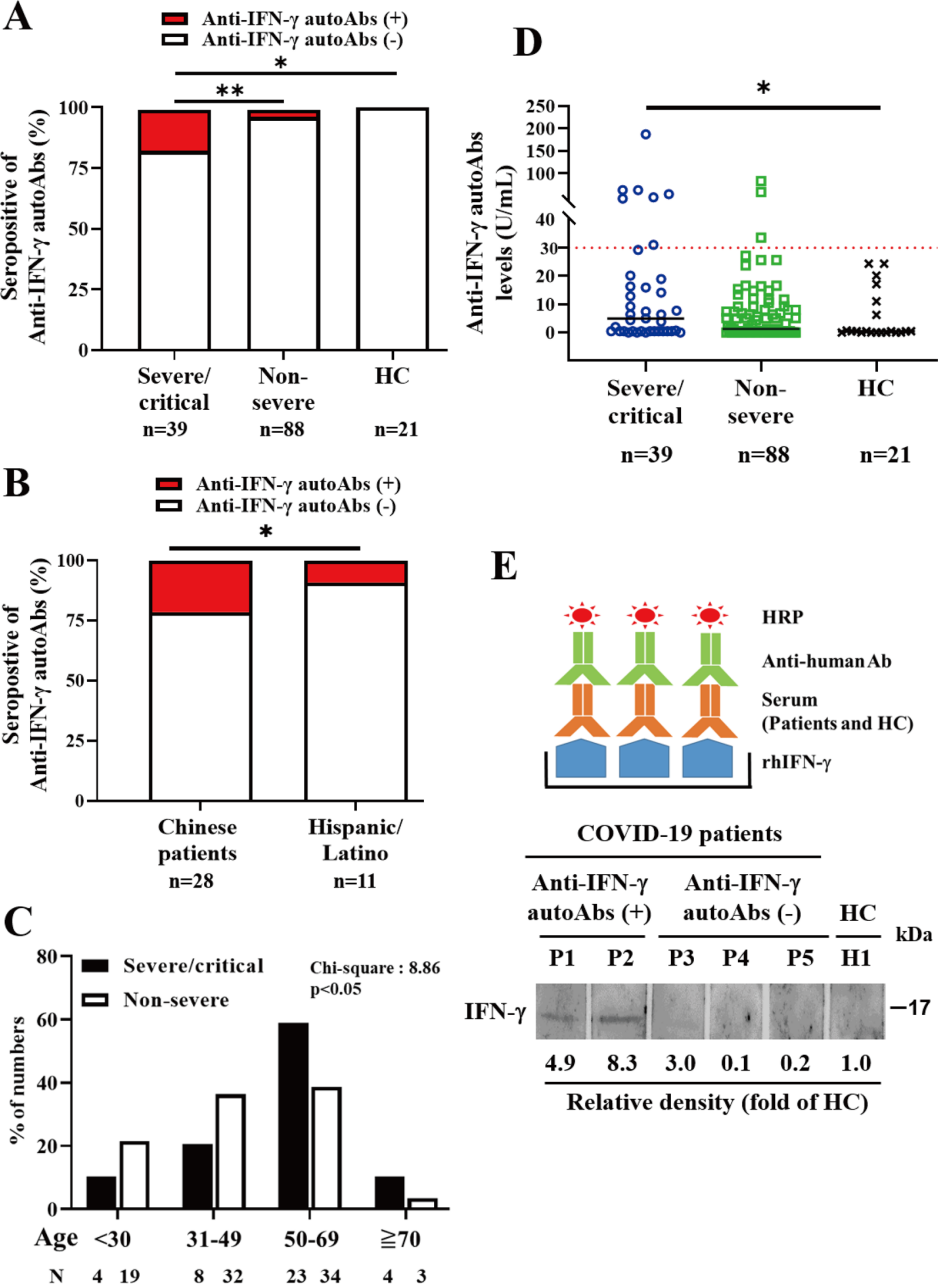
Comparison of the proportion of positive anti-IFN-γ autoAbs. **a** Among the severe/critical COVID-19, non-severe COVID-19 patients, and healthy control (HC) subjects, or **b** between the Chinese and Hispanic/Latino groups in severe/critical COVID-19. *p* value determined by Chi-square text, *p < 0.05, **p < 0.01. **c** Age discrimination in quartiles, the third and fourth quartile of age (≧ 50 years) still had a higher proportion of anti-IFN-γ autoAbs compared with other quartiles. *p*-value determined by Chi-square (CHIDIST), *p < 0.05. **d** Comparison of anti-IFN-γ auto-Abs titers among three groups of subjects. The horizontal line indicates the median value for each group respectively. The horizontal line in red color indicates the cut-off value of seropositivity for serum anti-IFN-γ autoAbs. *p value < 0.05, versus HC, determined by the Kruskal–Wallis test using a post-hoc Dunn’s test. **e** Schematic diagram of immunoblotting for confirming the presence of anti-IFN-γ autoAb. Immunoblots presented the binding activity of anti-IFN-γ autoAbs in the tested serum sample from COVID-19 patients or HC

As illustrated in Fig. 1a, the severe/critical COVID-19 patients had a significantly higher proportion of positive anti-IFN-γ autoAbs (7/39, 18.0%) than non-severe patients (3.4%, *p* <0.01) or HC subjects (0.0%, *p* <0.05). A higher proportion of positive anti-IFN-γ autoAbs was also found in older patients (defined as ≧ 50 years) (80.0%) and male patients (70.0%) when compared with autoAbs-negative patients (53.0% and 48.7%, respectively). Besides, Chinese patients with severe/critical COVID-19 (6/28, 21.4%) had a significantly higher proportion of positive autoAbs than the Hispanic/Latino cohort (1/11, 9.1%, *p* <0.05) (Fig. 1b). With the age discrimination in quartiles, the third and fourth quartile of age (≧ 50 years) still had a higher proportion of anti-IFN-γ autoAbs compared with other quartiles (*p* <0.05) (Fig. 1c).

As shown in Fig. 1d, patients with severe/critical COVID-19 had higher titers of anti-IFN-γ autoAbs (median, 5.01 U/ml, IQR 0.44–18.93 U/ml) than non-severe COVID-19 patients (1.33 U/ml, IQR 0.06–8.05 U/ml, *p* = 0.11) or HC subjects (0.44 U/ml, IQR 0.04–8.66U/ml, *p* <0.05).

Similar to the data from the ELISA-based assay, the immunoblotting assay confirmed the presence of serum anti-IFN-γ autoAbs in severe COVID-19 patients with positive autoAbs (P1 and P2), but not in COVID-19 patients with negative autoAbs (P3, P4, and P5) or in healthy control participants (H1) (Fig. 1e).

### Effective neutralization of IFN-γ signaling with anti-IFN-γ autoAbs in vitro

**Fig. 2:**
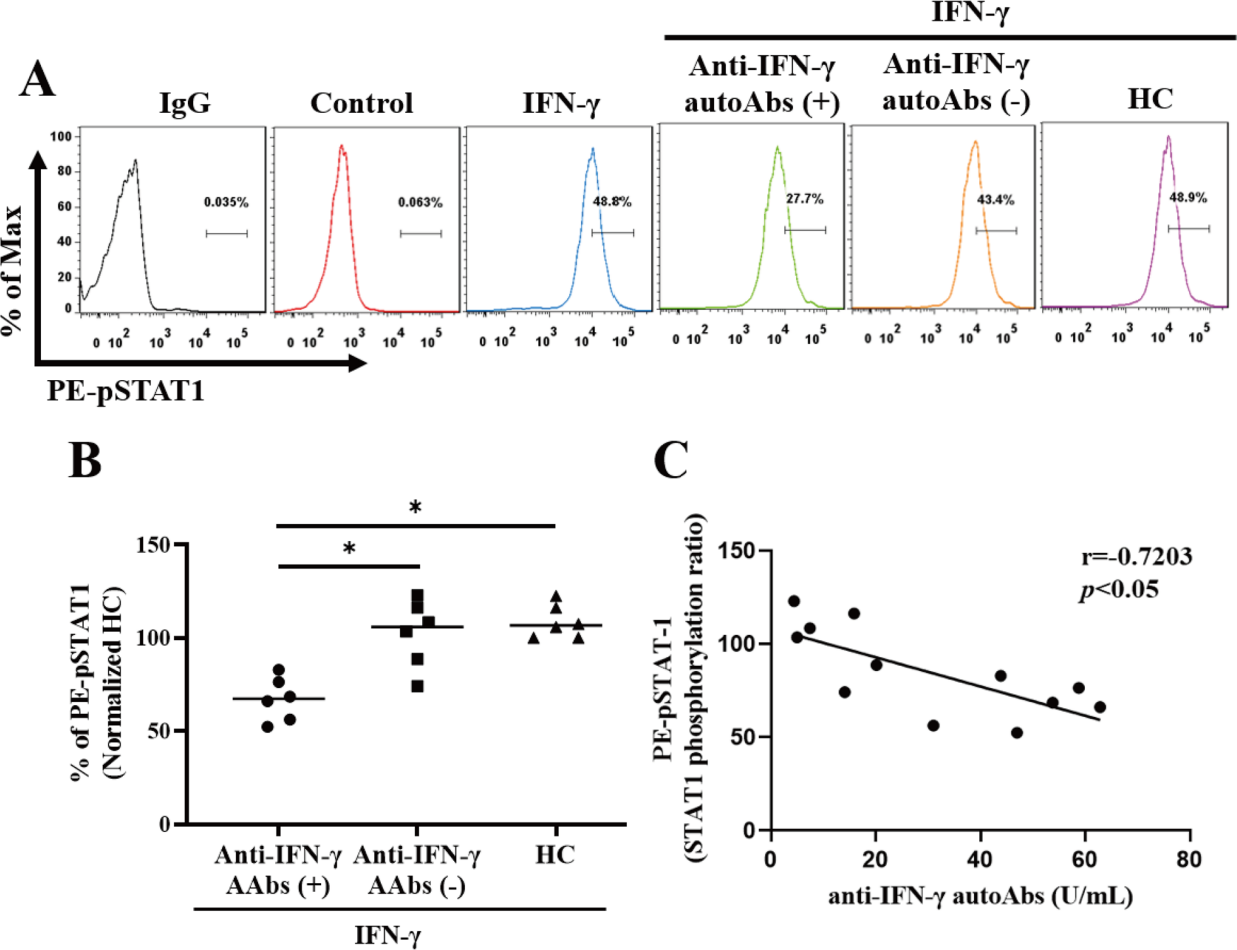
Effective neutralization of IFN-γ signaling with anti-IFN-γ autoAbs in vitro. The inhibition of IFN-γ-induced STAT1 phosphorylation in THP-1 cells treated with serum samples from anti-IFN-γ-autoAb-positive, anti-IFN-γ-autoAb-negative patients or HC subjects. **a** The representative histograms of the inhibition of IFN-γ-induced PE (phycoerythrin)-STAT1 phosphorylation in THP-1 cells treated with serum samples from anti-IFN-γ-autoAb-positive, anti-IFN-γ-autoAb-negative patients, or HC subjects. **b** Comparison of pSTAT1 proportion after inhibition with serum from three groups of subjects.** c** Anti-IFN-γ-autoAb levels were negatively associated with STAT-1 phosphorylation (ratio). Spearman’s nonparametric test was used in the correlation analysis. Data are presented as a logarithmic scale and box-plot diagrams, with the box encompassing the 25th percentile (lower bar) to the 75th percentile (upper bar). The horizontal line within the box indicates the median value respectively for each group. *p value < 0.05, ***p value < 0.001, determined by the Kruskal–Wallis test with a post-hoc Dunn’s test

The blocking effect of anti-IFN autoantibodies on IFN-induced STAT1 phosphorylation and its signal transduction were assessed by flow cytometry analysis as reported in other studies [20–22]. To evaluate the baseline values of fluorescence intensity, we determined the phosphorylation of STAT1 in unstimulated THP-1 cells treated with sera from patients with or without anti-IFN-γ autoAbs, and HC, respectively. There was no increase in the phosphorylation of STAT1 in unstimulated THP-1 cells treated with sera from each group (Additional file 1: Fig. S1). After adjustment for STAT1 phosphorylation of unstimulated THP-1, the serum samples from the autoAbs-positive patients could more effectively suppress the STAT1 phosphorylation (median 67.28%, IQR 55.4–78.0%) than those from the autoAbs-negative patients (median 105.9%, IQR 85.5–78.0%, *p* <0.05) or HC (median 106.6%, IQR 100.0–117.8%, *p* <0.05) (Fig. 2a, b). Besides, there was an inverse correlation between the STAT1-phosphorylation ratio in stimulated THP-1 cells treated with sera and the titers of anti-IFN-γ autoAbs (r = − 0.720, *p* <0.05) (Fig. 2c).

### Differences in serum cytokines levels between COVID-19 patients and HC

**Fig. 3:**
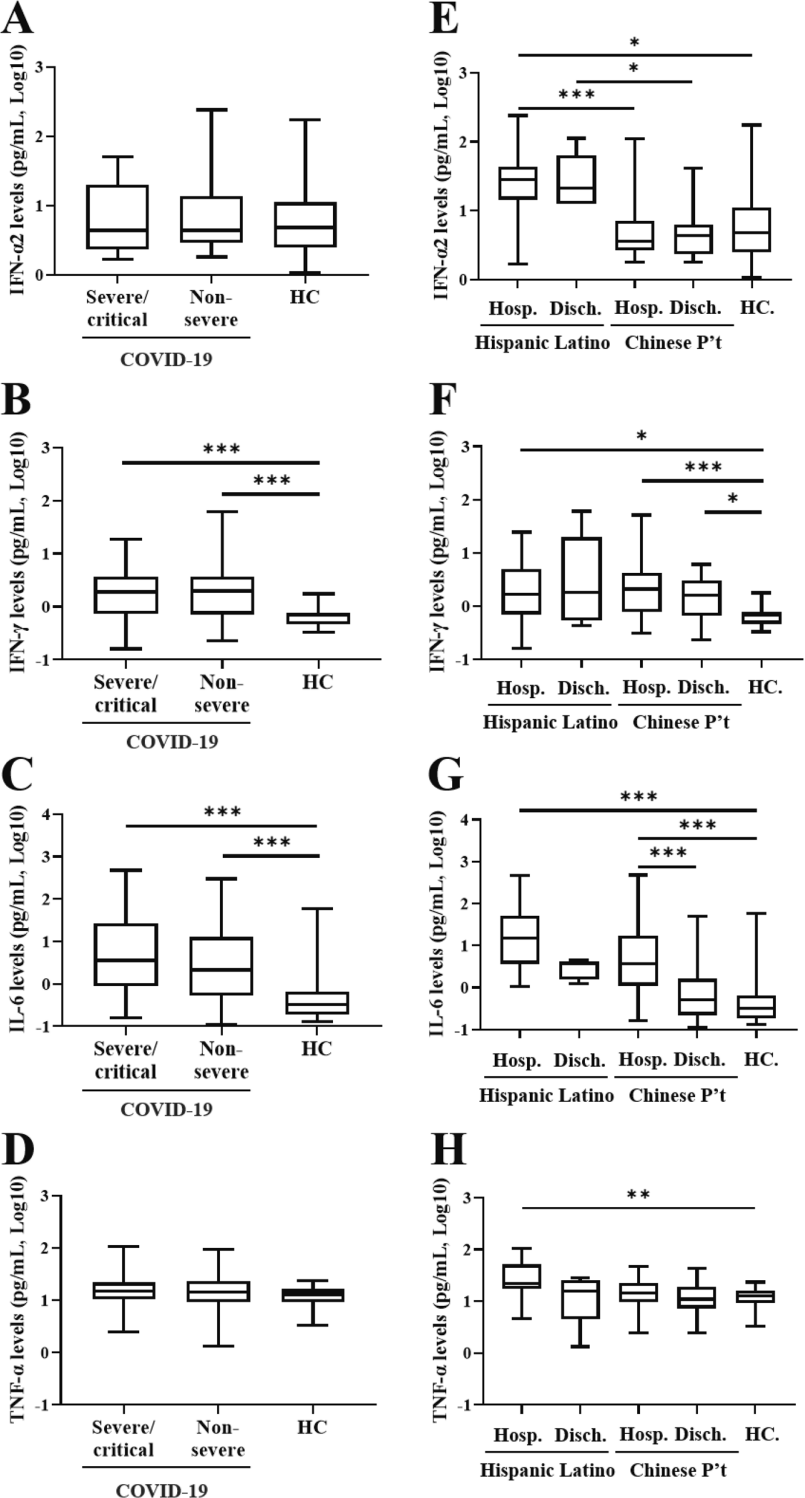
Comparison of serum levels of cytokine profiles among severe/critical COVID-19, non-severe COVID-19, and HC subjects. The differences in the levels of **a** IFN-α2, **b** IFN-γ, **c** IL-6, and **d** TNF-α among severe/critical COVID-19, non-severe COVID-19, and healthy control (HC). The differences in the levels of **e** IFN-α2, **f** IFN-γ, **g** IL-6, and **h** TNF-αbetween the different time points of blood sampling in the “severe” and “non-severe” groups respectively. COVID-19: coronavirus disease 2019; IFN: interferon; IL: interleukin; TNF-α: tumor necrosis factor-α. Data are presented as a logarithmic scale and box-plot diagrams, with the box encompassing the 25th percentile (lower bar) to the 75th percentile (upper bar). The horizontal line within the box indicates the median value respectively for each group. *p value < 0.05, ***p value < 0.001, determined by the Kruskal-Wallis test with a post-hoc Dunn’s test

As illustrated in Fig. 3b, c and Table 1, significantly higher serum levels of IFN-γ and IL-6 were observed COVID-19 patients, irrespective to the severity, compared with HC participants. Comparing severe/critical and non-severe COVID-19 patients only, there were no significant differences in serum levels of IFN-α, IFN-γ, IL-6, or TNF-α. There were also no significant differences in serum levels of IFN-α or TNF-α between COVID-19 patients, irrespective to the severity, and HC participants.

Besides, we analyzed the differences in serum cytokine levels at different time points of blood sampling in the “severe” and “non-severe” groups respectively. At the time of hospital admission, the severe/critical COVID-19 patients had significantly higher levels of IL-6 (median, 11.7 pg/mL, IQR 3.5-49.4 pg/mL) than non-severe COVID-19 patients (2.1 pg/mL, IQR 0.5-12.8 pg/mL, *p* <0.05, Fig. 3g). Among the severe/critical COVID-19 patients, significantly higher levels of IL-6 were also observed in blood samples obtained at the time of hospital admission compared with those obtained at the time of discharge (0.9 pg/mL, IQR 0.3-1.3 pg/mL, *p* <0.001, Fig. 3g).

Among all COVID-19 patients, significantly higher levels of IFN-α, IL-6, or TNF-α were shown in serum samples from the Hispanic patients compared with those from the Chinese patients (Additional file 2: Table S1).

### Logistic regression analysis to identify predictive markers for severe/critical COVID-19

Various inflammatory cytokines, such as interleukin IL-6, IFN-γ, and tumor necrosis factor-α, are elevated in patients with severe COVID-19 [23, 24]. Several previous systematic reviews and meta-analyses of studies have shown that cytokine storms are associated with the severity of COVID-19 [25, 26]. Thus, we enrolled the major inflammatory cytokines, demographic data, and anti-IFN-γ autoAbs in the logistic regression analysis to identify the potential markers for predicting severe/critical COVID-19. As illustrated in Table 2, the multivariate regression analysis identified age and anti-IFN-γ autoAbs titers as the significant predictors of severe/critical COVID-19. Old age (≧ 50 years) and positivity for anti-IFN-γ autoAbs could also significantly predict the severity of COVID-19 (odds ratio, 3.07, 95% CI 1.32-7.16, *p* <0.05; 6.84, 95% CI 1.53-30.64, *p* <0.05; respectively). However, there was no significant correlation between age and anti-IFN-γ autoAbs titers in the severe/critical COVID-19 patients or in all COVID-19 patients.

**Table 2:** Logistic regression analysis of demographic data, anti-IFN-γ autoAbs, and cytokine profiles for predicting severe/critical COVID-19 patients in the studied cohort

Baseline variables	Univariate model			Multivariate model		
	OR	95% CI	*p* value	OR	95% CI	*p* value
Age, ≧ 50 years	3.10	(1.39–6.91)	0.006	2.78	(1.22–6.37)	0.015
*Gender*						
Female	Ref					
Male	0.80	(0.38–1.71)	0.563			
Anti-IFN-γ Ab titer	6.20	(1.51–25.4)	0.011	5.67	(1.33–24.18)	0.019
IFN-α2 level, pg/ml	0.43	(0.18–1.01)	0.053			
IFN-γ level, pg/ml	0.94	(0.38–2.33)	0.907			
IL-6 level, pg/ml	1.20	(0.52–2.77)	0.658			
TNF-αlevel, pg/ml	1.84	(0.75–4.52)	0.182			

Variables in multivariate model (Forward: Conditional): age, gender, positivity, or titer of anti-IFN-γ Ab, IFN-α2, IFN-γ, IL-6, and TNF-α. Cut-off value: IFN-α2 (2.76 pg/ml), IFN-r (0.70 pg/ml), IL-6 (0.78 pg/ml), TNF-α (10.01 pg/ml)

OR: Odds ratio; 95% CI: 95% confidence interval; IFN: interferon; IL: interleukin; TNF-α: tumor necrosis factor-α

## Discussion

The severity of COVID-19 is associated with multiple factors, including age, preexisting comorbidities, and host immune status [27]. Mathew et al. recently revealed innate immunity to SARS-CoV-2 infection as a critical determinant of clinical outcome [28]. Accumulating evidence also indicates a crucial role of IFN-mediated immunity in controlling SARS-CoV-2 infection and COVID-19 severity [29, 30]. The presence of neutralizing autoAbs against IFNs-I has been shown in severe or life-threatening COVID-19 [16, 17]. AutoAbs against IFN-II (anti-IFN-γ autoAbs) are associated with AOID [3–10], yet the relation between anti-IFN-γ autoAbs and COVID-19 severity has not been explored. Herein, we are the first to demonstrate a significantly higher prevalence of anti-IFN-γ autoAbs in severe/critical COVID-19 patients compared with non-severe patients or HC participants. The anti-IFN-γ autoAbs could effectively block STAT1 phosphorylation and neutralize IFN-γ-mediated signaling. Besides, the multivariable logistic regression analysis revealed both the positivity and titers of anti-IFN-γ autoAbs as the significant predictors of severe/critical COVID-19. These findings suggest that anti-IFN-γ autoAbs are functional and may be associated with the severity of COVID-19.

The interplay between innate and adaptive immunity seems crucial in determining COVID-19 disease outcome. IFNs act as an important link between the innate and adaptive immune responses to viral infections. Ruetsch et al. revealed functional exhaustion of both types of IFN (IFN-α and IFN-γ) production, which have antiviral activities [31], in patients with severe COVID-19 [32]. Increasing evidence also indicates that the presence of neutralizing autoAbs to IFNs-I and an impaired IFNs-I-mediated immune response is related to severe clinical outcome of COVID-19 [16, 17, 33–36]. Recently, Chang et al. revealed the presence of autoAbs against IFN-II, anti-IFN-γ autoAbs, in four hospitalized patients with COVID-19 at baseline, which showed little change over time [17]. In our study, anti-IFN-γ autoAbs were detectable in the COVID-19 patients from two independent cohorts, with a combined prevalence of 7.9% among all patients. Given that the anti-IFN-γ autoAbs were detectable by using both ELISA and immunoblotting assays, our data provide robust evidence of the presence of autoAbs against IFN-II in COVID-19 patients. Besides, the severe/critical COVID-19 patients had significantly higher positive rate and higher titers of anti-IFN-γ autoAbs compared with non-severe COVID-19 patients. It is clinically significant that high-titer anti-IFN-γ autoAbs in COVID-19 patients may reflect increased severity of this disease. Detecting anti-IFN-γ autoAbs would help stratify the risk of severe clinical outcomes and promote potential therapeutic strategies for COVID-19 patients, such as recombinant IFN-γ therapy [37, 38].

Immune aging is characterized by enhanced self-reactivity and immunodeficiency [39]. Interestingly, the old-age (older than 50 years) group had a higher proportion of positivity for anti-IFN-γ autoAbs and more severe COVID-19 in our study. A previous study similarly revealed an increased prevalence of autoantibodies in the elderly without autoimmune diseases [26]. Given that we did not investigate anti-IFN-γ autoAbs in the elderly healthy control before SARS-CoV-2 infection for comparison, our findings need to be further validated. Besides, the higher prevalence of anti-IFN-γ autoAbs among the Chinese COVID-19 patients compared with the Hispanic patients (21.4% vs. 9.1%) in our study supports the previous findings that anti-IFN-γ autoAbs-associated AOID was prevalent in the Asian population [3–9]. Given that the genetic factors likely contribute to the substantial production of anti-IFN-γ autoAbs [40, 41], Chinese patients with severe/critical COVID-19/ had a significantly higher proportion of positive autoAbs than the Hispanic/Latino cohort.

To validate that anti-IFN-γ autoAbs are a cause and not a consequence of severe/critical COVID-19, we performed a flow cytometry analysis and revealed a neutralizing capacity of these auto-Abs through blocking STAT1 phosphorylation. Our results supported a functional blockade of IFN-γ-mediated antimicrobial immunity by anti-IFN-γ autoAbs in macrophages [42], and resonated with the report by Kim MH, et al. showing the compromised IFN-γ responses in critical COVID-19 patients [43]. These observations indicate that anti-IFN-γ autoAbs may potentiate the severity of COVID-19 through functional neutralization of the IFN-γ-mediated signaling pathway. Although functional characterization of anti-IFN-γ autoantibodies is limited only to a standard assay of IFN-γ-induced STAT1 phosphorylation, Chen et al. revealed that anti-IFN-γ autoantibodies could inhibit the phosphorylation of STAT1 and STAT3 in patients with *Talaromyces marneffei* infection [44].

Exaggerated innate and adaptive immune responses and inflammatory cytokines overproduction would develop in response to SARS-COV-2 infection [45, 46]. Our result also showed significantly higher levels of IL-6 and IFN-γ were observed in COVID-19 patients than in HC subjects. At the acute infection phase, severe/critical COVID-19 patients had significantly higher IL-6 levels than non-severe patients, suggesting a pathogenic role of IL-6 in a cytokine storm. An aberrant adaptive immune response against SARS-COV-2 may induce autoantibody formation [13–15], including anti-IFN-γ autoAbs. Gathering the evidence from other previous studies [20, 42, 43] and ours, we hypothesize that anti-IFN-γ autoAbs may amplify viral proliferation and raise a cytokine storm by neutralizing the IFN-γ-mediated signaling. The impairment of IFNs activity could exacerbate the inflammatory responses in severe COVID-19 patients [25, 47], which may provide a rationale for the use of IFNs in combination with anti-inflammatory agents in treating severe COVID-19 disease [48].

Accumulative evidence indicates that the neutralizing anti-IFN-γ autoAbs are recognized as a cause of AOID and associated with increased risks of opportunistic infections (OIs) [3–9]. The anti-IFN-γ autoAbs-positive patients usually have undetectable or low IFN-γ levels, which contribute OIs. However, two severe/critical COVID-19 patients with anti-IFN-γ autoantibodies also have detectable IFN-γ levels (8.11 and 10.08pg/ml). Possible contributing factors include an excessive inflammation with high IFN-γ levels triggered by SARS-CoV-2, a functional neutralization of the anti-IFN-γ autoantibodies through binding to the different domains of IFN-γ [20], and the possibility of false positive results of the used assay [49].

Despite the novel findings, there are some limitations in this study. The lack of a significant difference in the anti-IFN-γ autoAbs titers or cytokines levels between severe/critical and non-severe COVID-19 patients might be due to the small sample size. We were unable to identify the absolute anti-IFN-γ autoAbs titers that could predict severe/critical COVID-19, probably due to the insufficient number of autoAbs-positive patients. Because of the difficulties in obtaining specimens, we did not investigate anti-IFN-γ autoantibody titers in healthy Hispanic Latinos as another healthy control for more comparison. We will strive to conduct this investigation in future studies. The timing of blood collection from COVID-19 patients may not be during the acute infection phase. Given the different COVID-19 disease severity associated with different variants, the results regarding anti-IFN-γ autoAbs in our study may not be applied to infection with other variants of SARS-CoV-2. Moreover, we considered only the IFN-γ-induced phosphorylation of STAT1, and the possible effects on STAT3 remain to be explored. Given that healthy control participants were not sex and ethnicity matched with COVID-19 patients, the findings shown in our study await more external validation. Therefore, a large-scale study with sufficient statistical power is needed to validate the findings and further explore the biological function of anti-IFN-γ autoAbs in COVID-19 for more clinical implementation.

## Conclusion

Overall, we revealed that neutralizing anti-IFN-γ autoAbs were detectable in the serum of 7.9% of COVID-19 patients, even higher in severe/critical patients in the Asian population, up to 18.0%. Our results would add COVID-19 to the list of diseases with the presence of neutralizing anti-IFN-γ autoAbs. The detectable anti-IFN-γ autoAbs may be associated with COVID-19 severity through their functional blockade effects on IFN-γ-mediated STAT1-phosphorylation.

## Supplementary Information

**Additional file 1**. No increase in the phosphorylation of STAT1 on unstimulated THP1 cells treated with sera from the patients and HC.

**Additional file 2**. Demographic data and laboratory findings in the Hispanic Latino or Chinese patients with COVID-19 and Chinese healthy control participants.

## Data Availability

The datasets used and/or analyzed during the current study are available from the corresponding author on reasonable request.

## Abbreviations

AutoAbs: Autoantibodies
COVID-19: Coronavirus disease 2019
ELISA: Enzyme-linked immunosorbent assay
HC: Healthy control
IFN: Interferon
IFNR: Interferon receptor
ISGs: Interferon-stimulated genes
IL: Interleukin
OR: Odds ratio
NA: Not applicable
pSTAT1: Phosphorylated signal transducer and activator of transcription
SD: Standard deviation
SEM: The standard error of the mean
TNF-α: Tumor necrosis factor-α

## References

[1] Schoenborn JR, Wilson CB (2007). Regulation of interferon-gamma during innate and adaptive immune responses. Adv Immunol.

[2] Schroder K, Hertzog PJ, Ravasi T (2004). Interferon-gamma: an overview of signals, mechanisms and functions. J Leukoc Biol.

[3] Patel SY, Ding L, Brown MR (2005). Anti-IFN-gamma autoantibodies in disseminated nontuberculous mycobacterial infections. J Immunol.

[4] Wipasa J, Chaiwarith R, Chawansuntati K (2018). Characterization of anti-interferon-γ antibodies in HIV-negative immunodeficient patients infected with unusual intracellular microorganisms. Exp Biol Med (Maywood).

[5] Haverkamp MH, van Dissel JT, Holland SM (2006). Human host genetic factors in nontuberculous mycobacterial infection: lessons from single gene disorders affecting innate and adaptive immunity and lessons from molecular defects in interferon-gamma-dependent signaling. Microbes Infect.

[6] Browne SK, Burbelo PD, Chetchotisakd P (2012). Adult-onset immunodeficiency in Thailand and Taiwan. N Engl J Med.

[7] Chi CY, Lin CH, Ho MW (2016). Clinical manifestations, course, and outcome of patients with neutralizing anti-interferon-γ autoantibodies and disseminated nontuberculous mycobacterial infections. Medicine (Baltimore).

[8] Hase I, Morimoto K, Sakagami T (2017). Patient ethnicity and causative species determine the manifestations of anti-interferon-gamma autoantibody-associated nontuberculous mycobacterial disease: a review. Diagn Microbiol Infect Dis.

[9] Hanitsch LG, Lobel M, Muller-Redetzky H (2015). Late-onset disseminated Mycobacterium avium intracellulare complex infection (MAC), cerebral toxoplasmosis and salmonella sepsis in a German Caucasian patient with unusual anti-interferon-gamma IgG1 autoantibodies. J Clin Immunol.

[10] Hong GH, Ortega-Villa AM, Hunsberger S (2020). Natural history and evolution of anti-interferon-γ autoantibody-associated immunodeficiency syndrome in Thailand and the United States. Clin Infect Dis.

[11] Huang C, Wang Y, Li X, Ren L (2020). Clinical features of patients infected with 2019 novel coronavirus in Wuhan, China. Lancet.

[12] Mahajan S, Caraballo C, Li SX (2021). SARS-CoV-2 infection hospitalization rate and infection fatality rate among the non-congregate population in Connecticut. Am J Med.

[13] Rodríguez Y, Novelli L, Rojas M (2020). Autoinflammatory and autoimmune conditions at the crossroad of COVID-19. J Autoimmun.

[14] Tan KT, Hsu BC, Chen DY (2021). Autoimmune and rheumatic manifestations associated with COVID-19 in adults: an updated systematic review. Front Immunol.

[15] Dotan A, Muller S, Kanduc D (2021). The SARS-CoV-2 as an instrumental trigger of autoimmunity. Autoimmunity Rev.

[16] Bastard P, Rosen LB, Zhang Q (2020). Autoantibodies against type I IFNs in patients with life-threatening COVID-19. Science.

[17] Chang SE, Feng A, Meng W (2021). New-onset IgG autoantibodies in hospitalized patients with COVID-19. Nat Commun.

[18] Guan WJ, Ni ZY, Hu Y (2020). Clinical characteristics of coronavirus disease 2019 in China. N Engl J Med.

[19] Clinical Spectrum of SARS-CoV-2 Infection. https://www.covid19treatmentguidelines.nih.gov/overview/clinicalspectrum/. Accessed 31 Jan 2022.

[20] Krisnawati DI, Liu YC, Lee YJ (2019). Functional neutralization of anti-IFN-gamma autoantibody in patients with non-tuberculous mycobacteria infection. Sci Rep.

[21] Nithichanon A, Chetchotisakd P, Matsumura T (2020). Diagnosis of NTM active infection in lymphadenopathy patients with anti-interferon-gamma autoantibody using inhibitory ELISA vs. indirect ELISA. Sci Rep.

[22] Lin CH, Lewinski MK, Pache L (2016). Identification of a major epitope by anti-interferon-gamma autoantibodies in patients with mycobacterial disease. Nat Med.

[23] Bhaskar S, Sinha A, Banach M, Mittoo S, Weissert R, Kass JS (2020). Cytokine storm in COVID-19-immunopathological mechanisms, clinical considerations, and therapeutic approaches: The REPROGRAM Consortium Position Paper. Front Immunol.

[24] Tang Y, Liu J, Zhang D, Xu Z, Ji J, Wen C (2020). Cytokine storm in COVID-19: the current evidence and treatment strategies. Front Immunol.

[25] Chen R, Lan Z, Ye J (2021). Cytokine storm: the primary determinant for the pathophysiological evolution of COVID-19 deterioration. Front Immunol.

[26] Zawawi A, Naser AY, Alwafi H, Minshawi F (2021). Profile of circulatory cytokines and chemokines in human coronaviruses: a systemic review and meta-analysis. Front Immunol.

[27] Carlos WG, Dela Cruz CS, Cao B (2020). Novel Wuhan (2019-nCoV) coronavirus. Am J Respir Crit Care Med.

[28] Mathew D, Giles JR, Baxter AE (2020). Deep immune profiling of COVID-19 patients reveals distinct immunotypes with therapeutic implications. Science.

[29] Hadjadj J, Yatim N, Barnabei L (2020). Impaired type I interferon activity and inflammatory immune responses in severe COVID-19 patients. Science.

[30] Martin-Sancho L, Lewinski MK, Pache L (2021). Functional landscape of SARS-CoV-2 cellular restriction. Mol Cell.

[31] Bonjardim CA (2005). Interferons (IFNs) are key cytokines in both innate and adaptive antiviral immune responses—and viruses counteract IFN action. Microbes Infect.

[32] Ruetsch C, Brglez V, Crémoni M (2021). Functional exhaustion of both types of IFNs production in severe COVID-19 patients. Front Immunol.

[33] Bastard P, Gervais A, Le Voyere T (2021). Autoantibodies neutralizing type I IFNs are present in ~4% of uninfected individuals over 70 years old and account for ~20% of COVID-19 deaths. Sci Immunol.

[34] Goncalves D, Mezidi M, Bastard P (2021). Antibodies against type I interferon: detection and association with severe clinical outcome in COVID-19 patients. Clin Transl Immunol.

[35] Troya J, Bastard P, Planas-Serra L (2021). Neutralizing autoantibodies to type IFNs in >10% of patients with severe COVID-19 pneumonia hospitalized in Madrid, Spain. J Clin Immunol.

[36] Chauvineau-Grenier A, Bastard P, Servajea A, et al. Autoantibodies neutralizing type I interferons in 20% of COVID-19 deaths in a French hospital. RES Sq. 2021;rs.3.rs-915062. 10.1007/s10875-021-01203-3 PMC8791677 35083626

[37] Harada M, Furuhashi K, Karayama M (2021). Subcutaneous injection of interferon gamma therapy could be useful for anti-IFN-γ autoantibody associated disseminated nontuberculous mycobacterial infection. J Infect Chemother.

[38] Darazam IA, Shokouhi S, Pourhoseingholi MA (2021). Role of interferon therapy in severe COVID19: the COVIFERON randomized controlled trial. Sci Rep.

[39] Goronzy JJ, Li G, Yang Z (2013). The Janus head of T cell aging-autoimmunity and immunodeficiency. Front Immunol.

[40] Chi CY, Chu CC, Liu JP (2013). Anti-IFN-gamma autoantibodies in adults with disseminated nontuberculous mycobacterial infections are associated with HLA-DRB1*16:02 and HLA-DQB1*05:02 and the reactivation of latent varicella-zoster virus infection. Blood.

[41] Ku CL, Lin CH, Chang SW, Chu CC, Chan JF, Kong XF (2016). Anti-IFN-gamma autoantibodies are strongly associated with HLA-DR*15:02/16:02 and HLA-DQ*05:01/05:02 across Southeast Asia. J Allergy Clin Immunol.

[42] Krisnawati DI, Liu YC, Lee YJ (2019). Blockade effects of anti-interferon-(IFN-) γ autoantibodies on IFN-γ-regulated antimicrobial immunity. J Immunol Res.

[43] Kim MH, Salloum S, Wang JY (2021). Type I, II, and III interferon signatures correspond to COVID-19 disease severity. J Infect Dis.

[44] Chen ZM, Yang XY, Li ZT (2022). Anti-interferon-γ autoantibodies impair T-lymphocyte responses in patients with *Talaromyces marneffei* infection. Infect Drug Resist.

[45] De Biasi S, Meschiari M, Gibellini L (2020). Marked T cell activation, senescence, exhaustion and skewing towards TH17 in patients with COVID19 pneumonia. Nat Commun.

[46] Hu B, Huang S, Yin L (2021). The cytokine storm and COVID-19. J Med Virol.

[47] Blanco-Melo D, Nilsson-Payant BE, Liu W-C (2020). Imbalanced host response to SARS-CoV2 drives development of COVID-19. Cell.

[48] Hasselbalch HC, Skov V, Kjær L (2021). COVID-19 as a mediator of interferon deficiency and hyperinflammation: rationale for the use of JAK1/2 inhibitors in combination with interferon. Cytokine Growth Factor Rev.

[49] Bendtzen K (1998). Autoantibodies to cytokines. Eur J Clin Invest.

