# Supplementary material for: The detectable anti-interferon-γ autoantibodies in COVID-19 patients may be associated with disease severity
Source: Virol J. 2023 Feb 21;20:33. doi: 10.1186/s12985-023-01989-1 (PMC9942050; doi:10.1186/s12985-023-01989-1)
Supplement: Supplementary file 1 — Additional file 1. No increase in the phosphorylation of STAT1 on unstimulated THP1 cells treated with sera from the patients and HC. [file 12985_2023_1989_MOESM1_ESM.docx]

**
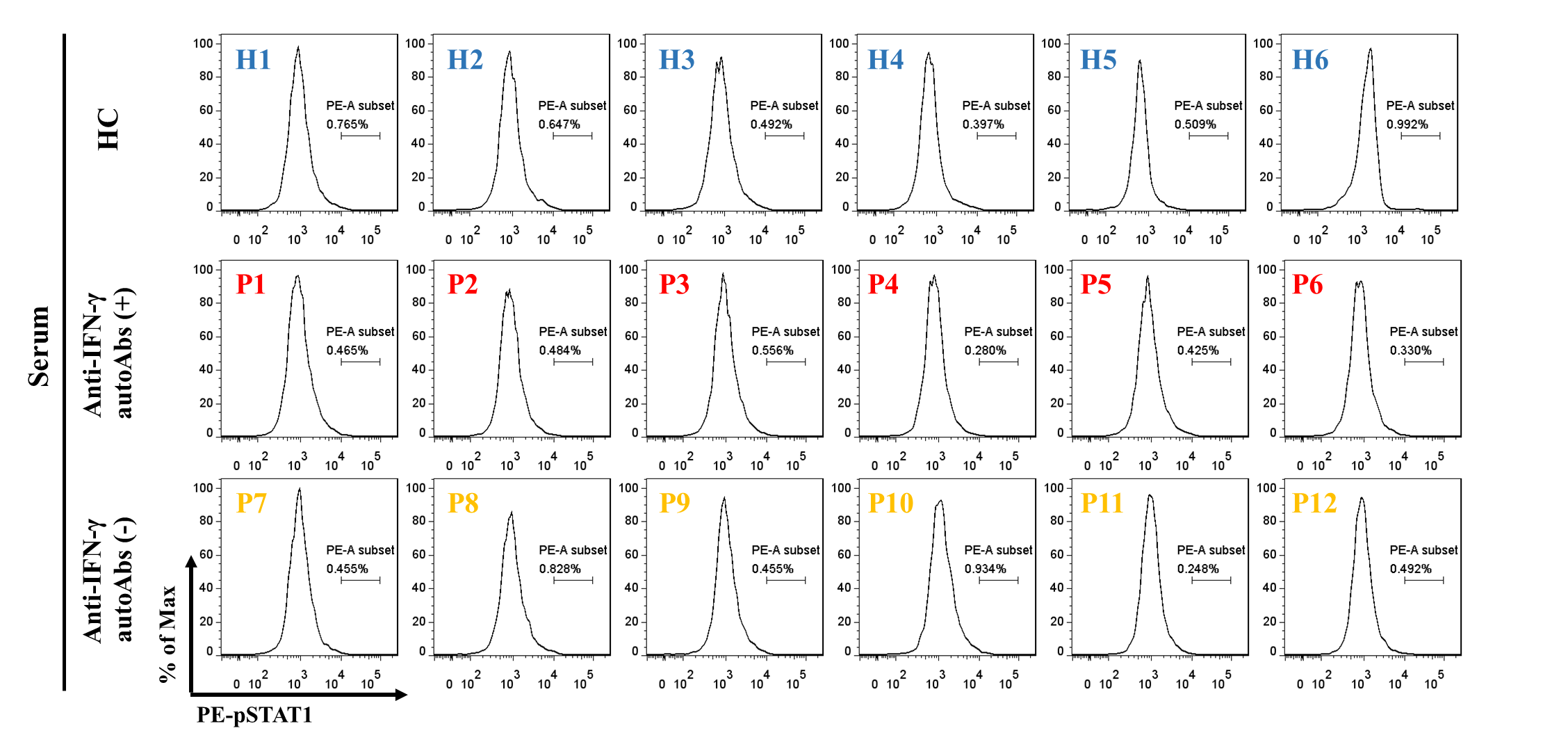
**

**Supplementary Fig. 1.** **No increase in the phosphorylation of STAT1 on unstimulated THP1 cells treated with sera from the patients and HC.** The STAT1 phosphorylation in THP-1 cells treated with serum samples from anti-IFN-γ-autoAb(+), anti-IFN-γ-autoAb(-) patients or HC subjects. The representative histograms of the inhibition of IFN-γ-induced PE (phycoerythrin)-STAT1 phosphorylation in THP-1 cells treated with serum samples from anti-IFN-γ-autoAb-positive, anti-IFN-γ-autoAb-negative patients, or HC subjects.
