# Supplementary material for: The detectable anti-interferon-γ autoantibodies in COVID-19 patients may be associated with disease severity
Source: Virol J. 2023 Feb 21;20:33. doi: 10.1186/s12985-023-01989-1 (PMC9942050; doi:10.1186/s12985-023-01989-1)
Supplement: Supplementary file 2 — Additional file 2. Demographic data and laboratory findings in the Hispanic Latino or Chinese patients with COVID-19 and Chinese healthy control participants. [file 12985_2023_1989_MOESM2_ESM.docx]

**Supplementary Table 1.** Demographic data and laboratory findings in the Hispanic Latino or Chinese patients with COVID-19 and Chinese healthy control participants

|  | Hispanic Latino  (n=25) | Chinese patients  (n=102) | Healthy control  (n=22) |
| --- | --- | --- | --- |
| Age at study entry, years | 53.01 ± 12.6 | 46.4 ± 16.9 | 41.9 ± 12.3 |
| Male proportion, n (%) | 16 (64.0%)^$$^ | 54 (52.9%)^$^ | 6 (27.3%) |
| Anti-IFN-γ autoAb (+), % | 2 (8.0%) | 8 (7.8%) | 0 (0.0%) |
| Anti-IFN-γ autoAb level, U/mL | 0.44 (0.4-3.3) | 4.71 (0.06-12.2) | 0.44 (0.04-8.7) |
| IFN-γ levels, pg/mL | 1.7 (0.6-5.4)^**^ | 2.0 (0.7-3.6)^***^ | 0.7 (0.5-0.8) |
| IFN-α2 levels, pg/mL | 24.1 (13.6-45.2) ^***,^**^###^** | 4.0 (2.5-6.3) | 4.8 (2.8-11.1) |
| IL-6 levels, pg/mL | 10.5 (3.6-25.7)^***,^**^##^** | 1.8 (0.5-7.9)^***^ | 0.3 (0.2-0.7) |
| TNF-α levels, ng/mL | 20.1 (15.3-37.9)^**,^**^##^** | 13.2 (9.1-20.9) | 12.9 (9.7-16.3) |

Data were expressed as mean ± SD, number (%), or median (25^th^ -75^th^ quartile range)

^**^p<0.01, ^***^p<0.001, vs. Health control, as determined by the Kruskal-Wallis test using a post-hoc Dunn’s test.

^##^p<0.01, ^###^p<0.001, vs. Chinese patients, as determined by the Kruskal-Wallis test using a post-hoc Dunn’s test.

^$^p<0.05, ^$$^p<0.01, vs. Health control, as determined by the Chi-square text.
